# Association between Pet Ownership and Obesity: A Systematic Review and Meta-Analysis

**DOI:** 10.3390/ijerph17103498

**Published:** 2020-05-17

**Authors:** Kenta Miyake, Kumiko Kito, Ayaka Kotemori, Kazuto Sasaki, Junpei Yamamoto, Yuko Otagiri, Miho Nagasawa, Sayaka Kuze-Arata, Kazutaka Mogi, Takefumi Kikusui, Junko Ishihara

**Affiliations:** 1 Department of Food and Life Science, School of Life and Environmental Science, Azabu University, 1-17-71 Fuchinobe, Chuo-ku, Sagamihara-city, Kanagawa 252-5201, Japan; k.miyake.088@gmail.com (K.M.); kotemori@azabu-u.ac.jp (A.K.); j-yamamoto@azabu-u.ac.jp (J.Y.); 2 Graduate School of Environmental Health, Azabu University, 1-17-71 Fuchinobe, Chuo-ku, Sagamihara-city, Kanagawa 252-5201, Japan; de1801@azabu-u.ac.jp; 3 Center for Human and Animal Symbiosis Science, Azabu University, 1-17-71 Fuchinobe, Chuo-ku, Sagamihara-city, Kanagawa 252-5201, Japan; nagasawa@azabu-u.ac.jp (M.N.); arata@azabu-u.ac.jp (S.K.-A.); mogik@azabu-u.ac.jp (K.M.); kikusui@azabu-u.ac.jp (T.K.); 4 Faculty of Nutritional Science, Sagami Women’s University, 2-1-1 Bunkyo, Minami-ku, Sagamihara-city, Kanagawa 252-0383, Japan; sasaki-k@star.sagami-wu.ac.jp; 5 Center for Science Information Services, Azabu University, 1-17-71 Fuchinobe, Chuo-ku, Sagamihara-city, Kanagawa 252-5201, Japan; otagiriy@azabu-u.ac.jp; 6 Department of Animal Science and Biotechnology, School of Veterinary Medicine, Azabu University, 1-17-71 Fuchinobe, Chuo-ku, Sagamihara-city, Kanagawa 252-5201, Japan

**Keywords:** pet ownership, companion animals, obesity

## Abstract

Obesity is a major risk factor for lifestyle-related diseases, including cardiovascular disease, type 2 diabetes, and hypertension. Several studies have investigated the association between pet ownership and obesity, but the findings have been inconsistent. This systematic literature review and meta-analysis assessed the association between pet ownership and obesity. Using PubMed and Scopus, we overviewed the literature published until December 2019 and selected pertinent data for meta-analysis. Two independent reviewers extracted the data. Pooled relative risks (RRs) with 95% confidence intervals (CIs) for obesity were calculated using the random-effects model with inverse-variance weighting. The 21 included articles were cross-sectional studies. Five publications (nine analyses) that reported adjusted RRs for BMI ≥ 25 were included in the meta-analysis. No significant association existed between pet ownership and obesity (pooled RR = 1.038; 95% CI, 0.922–1.167; I^2^ = 51.8%). After stratification by age group (children vs. adults), no significant association was detected (pooled RR = 0.844; 95% CI, 0.604–1.179; I^2^ = 64.1% vs. pooled RR = 1.099; 95% CI, 0.997–1.212; I^2^ = 25.2%). Similarly, no significant association was observed between dog ownership and obesity, indicating no association between pet ownership and obesity. However, no infer causation can be reported because all studies included in this meta-analysis were cross-sectional. Therefore, further prospective studies are needed.

## 1. Introduction

In recent years, the association between pet ownership and human health outcomes has been increasingly investigated. A survey of 22 countries, including Europe, the Americas, and Asia Pacific countries, reported that nearly half of the participants in the study were pet owners [1]. Keeping a pet has been associated with health-promoting effects, such as reduced risk of asthma [2] and allergies [3] among children. The frequency of annual doctor visits and medication administration is also reduced among pet owners, resulting in lower medical expenses [4]. Owning a dog is also associated with a 24% reduction in all-cause mortality [5], and reduced risk of cardiovascular disease [6]. However, there is some skepticism regarding the health benefits of pets; for example, a meta-analysis showing reduction of all-cause mortality included studies with insufficient confounding controls [5], and another meta-analysis reported inconsistent results. [7]

The prevalence of obesity, one of the risk factors of cardiovascular disease, is globally increasing. Approximately 39% of the global population aged 18 years and older have a body mass index (BMI) ≥25, and 13% of whom have a BMI ≥ 30 [8]. Obesity is also a risk factor for other lifestyle related diseases, such as hypertensions and type 2 diabetes [9].

The number of studies investigating the association between pet ownership and obesity has grown since 2000. However, the research findings have been inconsistent. Regarding the association between pet ownership and obesity in children, Timperio et al. reported that pet ownership meant a lower risk of obesity (OR = 0.5; 95% CI, 0.3–0.8) in a cross-sectional study in 2008 of 281 children aged 5–6 years [10], whereas Westgarth et al. reported that pet ownership was not associated with obesity (OR = 1.07; 95% CI, 0.86–1.34) in a cross-sectional study in 2012 of 6634 children aged 7 years [11]. Regarding adults, Timperio et al. reported that pet ownership was not associated with obesity (OR = 1.1; 95% CI, 0.9–1.5) in a cross-sectional study examination of 2000 adults in 2008. [10] In contrast, Parslow et al. mentioned that pet ownership was associated with adult obesity (OR = 1.16; 95% CI, 1.00–1.34) in a cross-sectional study of 5079 adults [12]. Therefore, we conducted a systematic review and meta-analysis, to incorporate all available previous studies, and to evaluate the association between pet ownership, including adults and/or children and obesity.

## 2. Materials and Methods

### 2.1. Search Strategy

Ιn this study, we performed a search in PubMed and Scopus from their inception until December 18, 2019. Studies evaluating the association between pet ownership and obesity were identified using a combination of the following keywords: “body mass index,” “obesity,” “body size,” “waist circumference,” “overweight,” “metabolic syndrome,” or “adipose tissue,” and “dog ownership,” “cat ownership,” “pet ownership,” “cat owner,” or “dog owner,” and “not obesity” or ”not veterinary.” The specific search strings for the databases are shown in Table S1. Two researchers independently searched for published studies through these databases. This study was conducted in accordance with the Preferred Reporting Studies for Systematic Reviews and Meta-Analyses (PRISMA) Statement checklist (Table S2).

### 2.2. Inclusion Criteria

For the systematic review, the inclusion criteria were set as follows: (1) exposure was having a pet (any kind of pet); (2) outcome indices were related with obesity (obesity, BMI, body weight, body shape, waist circumference, overweight, body fat, and metabolic syndrome); (3) participation of healthy individuals; (4) epidemiological studies; (5) language was restricted to English. Articles were excluded from the review when: (1) exposure was not pet ownership; (2) outcomes were unrelated to obesity; (3) participants were not community-dwelling people; (4) studies were involved in animal or cell studies, systematic reviews, meta-analysis, or conference reports.

The inclusion criteria for the meta-analysis were as follows: (1) observational or intervention studies with reported relative risks (RRs), i.e., hazard ratios, risk ratios, or odds ratios (ORs) with confidence intervals (CIs). Study eligibility was individually determined by KM and KS. Discrepancies in the determination of study eligibility were resolved by mutual consensus. The quality of each study was also independently evaluated by KM and KS using the Newcastle–Ottawa scale [13,14].

### 2.3. Data Extraction

After excluding the duplicates, the remaining publications were screened by the titles and review of their abstracts. Full-text assessment of the remaining studies served as a secondary screening, which was done in accordance with the selection criteria. Based on the secondary screening, publications that satisfied the selection criteria were chosen for a systematic review.

We used the standard data extraction form to obtain the characteristics of the individual studies. The form included the title, publication year, name of the first author, country of origin, study design, types of pets owned by the pet owners, number of participants, percentage of male participants, age of the participants, follow-up years, how to evaluate the outcomes, results related to the pet ownership and obesity (i.e., adjusted relative risk and their 95% CI), adjusted variables used in the multivariable analysis, and results of the quality assessment.

### 2.4. Statistical Analysis

Relative risk (95% CI) was used to summarize the association between pet ownership and the risk of obesity. We used a random-effects model with inverse-variance weighting to calculate the pooled RR (95% CI). Heterogeneity among the studies was identified using I^2^ statistics. An I^2^ value of 0–25%, 25–50%, 50–75%, and >75% indicated insignificant, low, moderate, and high heterogeneity, respectively. Stratified analysis was conducted by age group (adults aged ≥ 19 years vs. children) and dog owners. STATA 15.0 (Stata Corp, College Station, TX, USA) was used for meta-analysis.

## 3. Results

**Figure 1:**
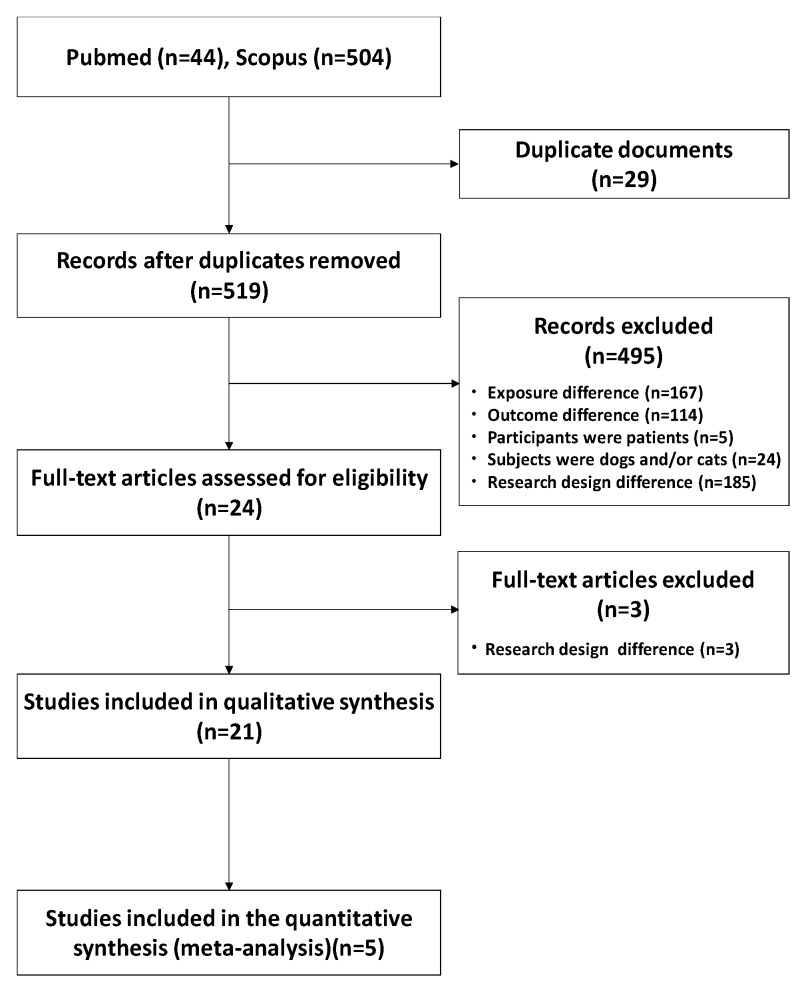
Flowchart of the literature review.

Figure 1 shows a flowchart of the article selection. After excluding 29 duplicates, 519 publications underwent title and abstract review. Of these, 24 articles underwent full-text review, and 21 articles [10,11,12,15,16,17,18,19,20,21,22,23,24,25,26,27,28,29,30,31,32] were included for a systematic review. Among the 21 publications, five articles that reported adjusted odds ratio and 95%CI were included in the meta-analysis [10,11,12,17,25]. As age-stratified analysis was included in these five articles, a total of nine analyses were used for the meta-analysis.

### 3.1. Characteristics of the Selected Articles (Systematic Review)

The characteristics of the selected articles are listed in Table 1. These studies were published from 1992 to 2019, mostly in Western countries; eight of them were conducted in the United States, six in Australia, three in the United Kingdom, one in Norway, one in Finland, one in Canada, and one in the Czech Republic. All of them were cross-sectional studies. When age-stratified analyses were included, there were 17 and eight analyses involving adults and children as participants, respectively. BMI was the most common evaluation method for the outcome. For analysis of the association between pet ownership and obesity, most of the studies compared the proportion of overweight (BMI ≥ 25) or obesity (BMI ≥ 30) (16 analyses). After considering confounding factors, most of the studies revealed the association using logistic regression analysis (nine analyses). Regarding the result of the association between pet ownership and overweight/obesity, three analyses reported that pet owners were more obese, 17 analyses found no association, and five analyses reported that pet owners were less obese. Eighteen of the 25 analyses reported results of the association between dog ownership and being overweight/obesity, where two, 13, and three analyses reported that the dog owners were more obese, had no association, and were less obese, respectively. The mean quality assessment score by the Newcastle–Ottawa scale was 6.3 for 21 articles.

### 3.2. Quantitative Summary (Meta-Analysis)

**Figure 2:**
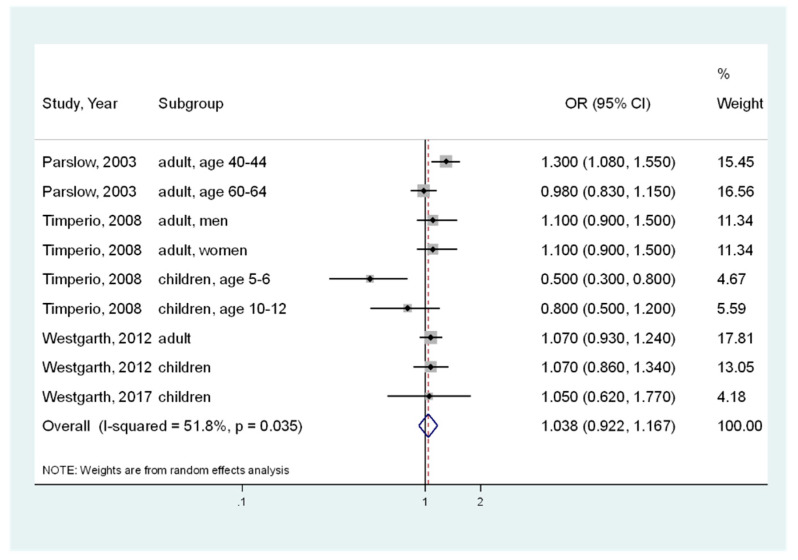
Overall meta-analysis of the association between pet ownership and obesity risk (body mass index ≥ 25). CI, Confidence Interval; OR, Odds Ratio.

In this study, a total of 24,555 participants from nine analyses in five articles were combined. No significant association between pet ownership and obesity was observed (Figure 2); the pooled OR was 1.038 (95% CI, 0.922–1.167) [10,11,12,17,25]. However, significant moderate heterogeneity was determined (I^2^ = 51.8%, *p* = 0.035).

**Figure 3:**
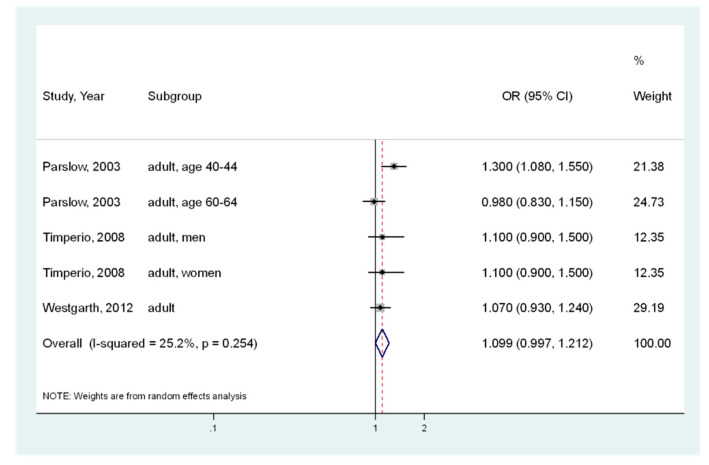
Meta-analysis of the association between pet ownership and obesity risk (body mass index ≥ 25) in adults. CI, Confidence Interval; OR, Odds Ratio.

**Figure 4:**
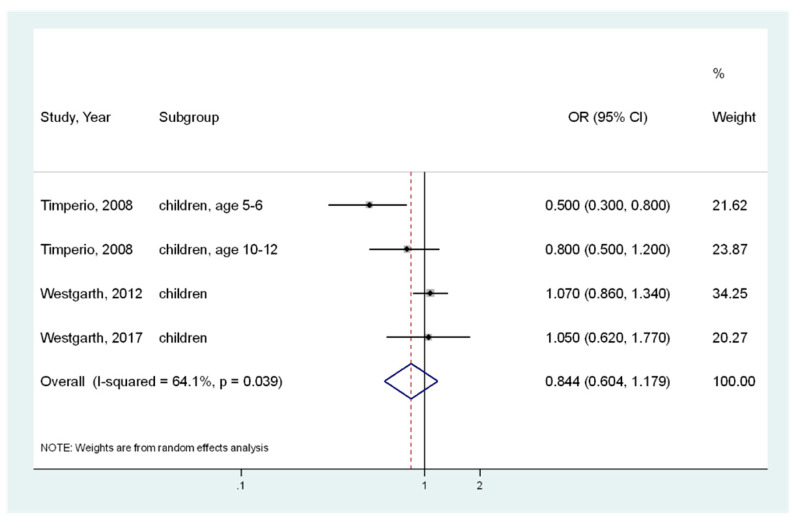
Meta-analysis of the association between pet (dog) ownership and obesity risk (body mass index ≥ 25) in children. CI, Confidence Interval; OR, Odds Ratio.

Assuming that the participants’ age affected heterogeneity, stratified analyses were also conducted for adults (in five analysis) or children (in four analysis). For adults (Figure 3), the pooled OR of the five analyses was 1.099 (95% CI, 0.997–1.212) [10,12,25], indicating that there was no significant association between pet ownership and obesity. Insignificant heterogeneity was observed (I^2^ = 25.2%, *p* = 0.254). For children (Figure 4), the pooled OR of the four analyses was 0.844 (95% CI, 0.604–1.179) [10,11,17], indicating no significant association. Significant moderate heterogeneity was indicated (I^2^ = 64.1%; *p* = 0.039).

**Figure 5:**
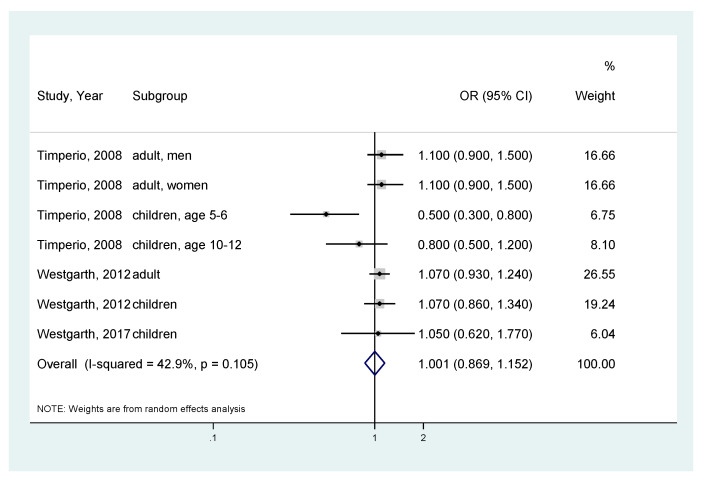
Meta-analysis of the association between dog ownership and obesity risk (body mass index ≥ 25). CI, Confidence Interval; OR, Odds Ratio.

**Figure 6:**
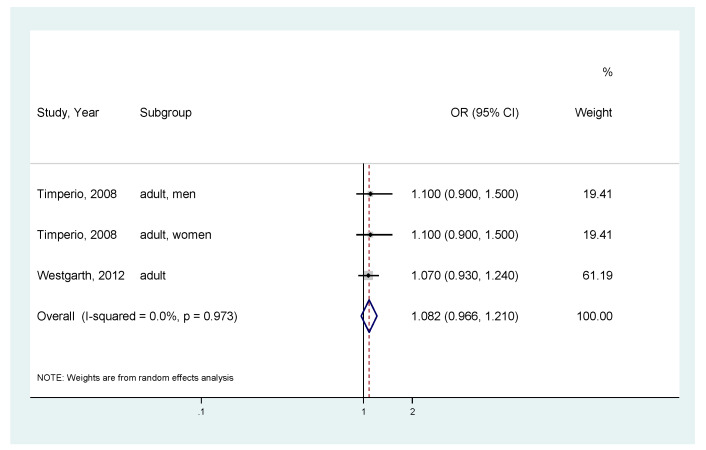
Meta-analysis of the association between dog ownership and obesity risk (body mass index ≥ 25) in adults. CI, Confidence Interval; OR, Odds Ratio.

Furthermore, we conducted a similar analysis focusing on dog owners because we suspected that dog-walking had a preventive effect on obesity. For all (Figure 5), there was no significant association between dog ownership and obesity (for seven analyses, the pooled OR = 1.001; 95% CI, 0.869–1.152; I^2^ = 42.9%; *p*-value for I^2^ = 0.105) [10,11,17,25]. Stratified by age group, no significant association was also observed between dog ownership and obesity. For adults, pooled analysis of three results showed the pooled OR = 1.082 (95% CI, 0.966–1.210; I^2^ = 0%; *p*-value for I^2^ = 0.973) (Figure 6) [10,25]. For children, the result is presented in Figure 4.

## 4. Discussion

We conducted a systematic review and meta-analysis to summarize published articles (until December 2019) that investigated the association between pet ownership and obesity. A total of 21 articles were included in the systematic review, five of which were included in the meta-analysis. There was no significant association between pet ownership and obesity regardless of age group. Furthermore, no significant association was observed between dog ownership and obesity.

The number of studies that directly investigated the association between pet ownership and obesity is limited, and only a few have reported RRs adjusted for confounding factors. Regarding physical activity, only one of the five articles was adjusted for this. Among the elderly, 17% of dog owners who walked at least three times or more per week were obese compared to 29% among dog owners who did not walk three times per week [31]. Additionally, 19% of non-dog owners who walked at least three times or more per week were obese compared to 25% of non-dog owners who did not walk at least three times per week. These results suggested an association between walking and obesity, regardless of dog ownership. However, many dog owners maintained their walking habits beyond 3 years [31]. The importance of distinguishing the daily amount of physical activity from an increase in the amount of physical activity associated with dog ownership is important in our analysis. The fact that the physical activity levels were not sufficiently evaluated in almost all of the studies included in the meta-analysis may have affected the findings.

Obesity is caused by an imbalance between energy intake and expenditure. According to the World Health Organization, one of the causes of obesity is the global increase in the consumption of energy-dense foods that are high in fat [8]. A study of 3185 dog owners in 11 European countries reported that increasing positive attitudes towards a healthy diet decreased the likelihood of being overweight/obese (β = −1.662, Standard error = 0.441, *p* < 0.001) [33]. Only one of the studies included in this meta-analysis considered the influence of diet [17]. However, the questionnaire used in this study only assessed the eating habits of participants, based on the recall of healthier/less healthy foods that were consumed on the previous day with yes/no options [17,34]. The questionnaire did not assess the nutritional intake. Therefore, further studies are needed to assess the quantitative dietary intake using tools, such as dietary records.

In our study, pet ownership was treated as an exposure. However, the information on the type of pets could not be obtained in some studies. A study by Garcia et al. reported that 64%, 68%, 63%, and 68% of the non-pet, dog, cat, and bird owners, respectively, had a BMI ≥ 25, indicating that the type of pet may be related to pet owners’ obesity [20]. In addition, although most of the studies included in the meta-analyses treated dog ownership as exposure, the dog breed (i.e., giant, large, medium, small, toy) was not considered. We conducted a meta-analysis only in dog owners but did not find any association between pet ownership and obesity. Therefore, pet type or pet breed may mask the association between pet ownership and obesity.

This study had several limitations. First, a limited number of studies were included in the meta-analysis. In the future, as the number of studies involving different populations increase, the null results reported in the present study may change. Second, as all the studies included in the meta-analyses were cross-sectional, the study design may have led to the null results in the present study. Therefore, prospective studies are needed to elucidate the association. Finally, as most of the included studies were conducted in Western countries, the results cannot be generalized to other populations, such as Asians and Africans.

## 5. Conclusions

Systematic review and meta-analysis did not suggest the preventive effect of pet ownership on obesity. However, as all studies extracted by the systematic review process were cross-sectional, a causal relationship cannot be inferred. Therefore, further prospective studies are needed.

## Figures and Tables

**Table 1:** Characteristics of the included studies in systematic review.

Reference	Title	Author, Year,	Country	Study Design	Pet Type	N (Pet Owner:Non-Pet Owner)	Percentage of Men (Pet Owner:Non-Pet Owner)	Mean Age (SD) (Pet Owner:Non-Pet Owner)	Outcome Measure	Findings	Adjusted Variables	Quality Assessment Selection (0–4 Stars) Comparability (0–2 Stars) Outcome (0–3 Stars)
[15]	Does Dog Ownership Affect Physical Activity, Sleep, and Self-Reported Health in Older Adults?	Mičková, E. et al., 2019	Czechia	Cross-sectional study	Dog	44 (26:18)	41	68(5.4):71(5.5)	BMI (Mean)	Mean (SD) of BMI in non-dog owners and dog owners, respectively; 28.8 (5.4), 26.1 (4.1); *p* = 0.0213	-	Selection (★) Comparability (-) Outcome (★★)
[16]	Pet ownership and the risk of dying from lung cancer, findings from an 18 year follow-up of a US national cohort	Adhikari, A. et al, 2019	United States	NHANESⅢ	Pet	13,725 (5902:7823)	48.4	43.3	BMI (%)	Pet owner’s BMI distribution in men and women, respectively; Underweight; 1.05%, 3.39% Normal weight; 37.71%, 40.62% Overweight; 39.95%, 27.71% Obese; 21.29%, 28.28% No significant differences were obwerved between pet owners and non-pet owners by chi-square test (*p* = 0.46 in men, *p* = 0.18 in women).	-	Selection (★★★★) Comparability (-) Outcome (★★)
[17]	The association between dog ownership or dog walking and fitness or weight status in childhood.	Westgarth, C. et al, 2017	United Kingdom	Cross-sectional study	Dog	798 (295:503)	-	9–10 years	BMI (OR)	Dog onwership and "overweight or obese"; Model 1; OR = 1.04 (95%CI; 0.67, 1.60) Model 2; OR = 1.05 (95%CI; 0.62, 1.77) Dog onwership and obese; Model 1; OR = 1.60 (95%CI; 0.80, 3.20) Model 2; OR=1.09 (95%CI; 0.43, 2.79)	Model 1; gender, developmental age, Index of Multiple Deprivation 2007Model 2; gender, developmental age, Index of Multiple Deprivation 2007, positive food score and negative food score	Selection (★★★) Comparability (★★) Outcome (★★★)
[18]	Interactions between Neighbourhood Urban Form and Socioeconomic Status and Their Associations with Anthropometric Measurements in Canadian Adults.	McCormack, G.R. et al, 2017	Canada	Cross-sectional study	Dog	851 (278:573)	37.6	52.8 (14.3)	Waist circumference (β) Waist-to-hip ratio (β) BMI (β)	Non-dog ownership and Waist circumference; Model 2; β = −0.02 (95%CI; −1.48, 1.45) Non-dog ownership and Waist-to-hip ratio; Model 2; β = −0.27 (95%CI; −1.77, 1.23) Non-dog ownership and BMI; Model 2; β = −0.05 (95%CI; −0.68, 0.59)	Model 2; neighbourhood street pattern and neighbourhood level socioeconomic status plus all sociodemographic and health variables	Selection (★★★★) Comparability (★★) Outcome (★★)
[19]	Dog Walking, the Human-Animal Bond and Older Adults’ Physical Health.	Curl, A.L. et al, 2017	United States	The Health and Retirement Study	Dog	771 (271:500)	51.66: 46.58	67.03 (10.64)	BMI (%)	Mean (SD) of BMI;Dog non-owners; 28.41 (0.33) Dog owners non-dog walking; 29.43 (0.64) Dog owners dog walking; 27.84(0.48) Multivariate results indicated that dog ownership was not associated with better physical health and health behaviors (data not shown).	age, household income, gender, race, ethnicity, years of education, and marital status.	Selection (★★★★) Comparability (-) Outcome (★)
[20]	Pet Ownership and Cancer Risk in the Women’s Health Initiative.	Garcia, D.O. et al., 2016	United States	Cross-sectional study	Dog, cat, bird	123,560 (41,607:81,953) Dog; 20,981 Cat; 19,288 Bird; 1338	0	Dog owner; 61.7 (7.0) Cat owner; 61.8 (7.1) Bird owner; 62.3 (7.4) No-pets; 64.1 (7.1)	BMI (Mean and %)	Mean (SD) of BMI in no pets, dog(s), cat(s), bird(s), respectively; 27.8 (5.8), 28.3 (6.0), 27.8 (6.0), 28.5 (6.1) BMI (%) in no pets, dog(s), cat(s), bird(s), respectively; <25; 36.0%, 32.6%, 37.2%, 32.0% 25-29.9; 35.1%, 34.9%, 33.9%, 34.1% ≥30; 28.9%, 32.6%, 28.9%, 33.8%	-	Selection (★★★★) Comparability (-) Outcome (★★)
[21]	Dog walking among adolescents: Correlates and contribution to physical activity	Engelberg, J.K. et al., 2016	United States	Cross-sectional study	Dog	925 (484:441)	47.1: 52.2	Dog owners;(non-walk: walk) 14.16 (1.45): 14.02 (1.39)Non-dogs; 14.12 (1.37)	BMI (CDC)	Mean (95% CI) CDC Age adjusted BMI Percentiles; Dog non-owners; 66.53 (63.48, 69.57) Dog owners non-dog walking; 64.86 (59.15, 68.90) Dog owners dog walking; 65.21 (61.64, 68.77)	adolescent age, gender, race/ethnicity, parent marital status, parent education and house type	Selection (★★) Comparability (★★) Outcome (★★)
[22]	Pet dogs and children’s health: Opportunities for chronic disease prevention?	Gadomski, A.M. et al, 2015	United States	Cross-sectional study	Dog	643 (470:133)	54.9: 54.9	6.72: 6.71	BMI (%)	BMI distribution in pet owners and non-pet owners, respectively; Nomal; 65.8%, 66.5% Overweight; 17.7%, 15.8% Obese; 16.6%, 17.7% No significant differences were obwerved between pet owners and non-pet owners by chi-square test (*p* = 0.80).	-	Selection (★★) Comparability (★★) Outcome (★)
[23]	Understanding the relationship between dog ownership and children’s physical activity and sedentary behaviour.	Christian, H. et al, 2012	Australia	Cross-sectional study	Dog	1218 (729:489)	47.7:48.5	11(0.8): 11(0.8)	BMI (%)	Overweight or obese BMI (%) in dog owners and non-dog owners, respectively; All children; 23.3, 23.3 Boys; 22.1, 23.3 Girls; 24.6, 23.3	-	Selection (★★★★) Comparability (-) Outcome (★★)
[24]	Health in older cat and dog owners: The Nord-Trondelag Health Study (HUNT)-3 study.	Enmarker, I. et al, 2012	Norway	Cross-sectional study	Dog, cat	12,297 (2358:9939)	51.4:44.7	65–101 years	BMI (Mean)	Mean (SD) of BMI in non-pet owners, cat owners, dog owners, respectively; 26.96 (5.69), 27.88 (4.88), 27.53 (4.50); *p* < 0.001	-	Selection (★★★★) Comparability (★★) Outcome (★★)
[10]	Is childhood obesity influenced by dog ownership? No cross-sectional or longitudinal evidence.	Westgarth, C. et al, 2012	United Kingdom	Cross-sectional study	Dog	6634 (1391:5243)	50.9	7 years	BMI (OR)	Dog ownership and overweight or obese; Crude; OR = 1.11 (95%CI; 0.95, 1.29) Model 1; OR = 1.11 (95%CI; 0.92, 1.35) Model 2; OR = 1.07 (95%CI; 0.86, 1.34) Dog ownership and obese; Crude; OR = 1.33 (95%CI; 1.08, 1.63) Model 1; OR = 1.30 (95%CI; 1.00, 1.70) Model 2; OR = 1.18 (95%CI; 0.88, 1.59)	Model 1: concurrent ownership of bird, fish, ‘other’ pet, number of people in household, presence of an older sibling, maternal social class, paternal social class, paternal education, maternal age at delivery, house type, whether mother owned pets as a child Model 2: Model 1 + maternal education, maternal social class, maternal smoking during pregnancy, parental obesity, gender of child, birth weight, TV watching at 38 months, sleep duration at 30 months	Selection (★★★) Comparability (★★) Outcome (★★★)
[25]	Dog ownership during pregnancy, maternal activity, and obesity: a cross-sectional study.	Westgarth, C. et al, 2012	United Kingdom	Cross-sectional study	Dog	13,215 (7670:5545)	0	Adult	BMI (OR)	Dog ownership and overweight or obese; Crude; OR = 1.18 (95%CI; 1.06, 1.30) Adjusted; OR = 1.07 (95%CI; 0.93, 1.24) Dog ownership and obese; Crude; OR = 1.31 (95%CI; 1.10, 1.57) Adjusted; OR = 0.97 (95%CI; 0.74, 1.27)	Adjusted:maternal education, maternal social class, mother worked during pregnancy, maternal age at delivery, previous living children, number of people in household, house type, mother had pets as a child.	Selection (★★★★) Comparability (★★) Outcome (★★)
[26]	The influence of the built environment, social environment and health behaviors on body mass index. results from RESIDE.	Christian, H. et al, 2011	Australia	Cross-sectional study	Dog	1551 (682 869)	41	40 (11.7)	BMI (β)	Dog ownership and BMI; β = −0.085 (*p* = 0.722)	Significant socio-demographic variables (sex, age, education, work hours per week, children at home, number of adults living in the house).	Selection (★★) Comparability (★★) Outcome (★★)
[27]	Pet ownership and adolescent health: cross-sectional population study.	Mathers, M. et al, 2010	Australia	Cross-sectional study	Pet	928 (823:105)	50.2	15.9 (1.2)	BMI (%)	BMI distribution in pet owners and non-pet owners, respectively; Nomal; 74.1%, 72.4% Overweight; 20.4%, 17.1% Obese; 5.5%, 10.5%	-	Selection (★★★★) Comparability (★★) Outcome (★★)
[28]	Physical activity, weight status, and neighborhood characteristics of dog walkers.	Coleman, K.J. et al, 2008	United States	Cross-sectional study	Dog	2199 (616:1583)	52	45 (11)	BMI (%)	BMI distribution in non-pet owners (NO), pet owners non-walkers (ONW), and pet owners walkers (OW), respectively; Overweight; 34%, 34%, 43% Obese; 22%, 28%, 17% Significant Differences; Overweight; NO<ONW<OWO bese; OW<NO<ONW	-	Selection (★★★) Comparability (-) Outcome (★★)
[11]a	Is dog ownership or dog walking associated with weight status in children and their parents?	Timperio, A. et al, 2008	Australia	Cross-sectional study	Dog	5–6 years; 281 10–12 years; 864	-	-	BMI (OR)	Dog ownership and overweight or obese; 5–6 years Model 1; OR = 0.7 (95%CI; 0.4, 1.4) Model 2; OR = 0.5 (95%CI; 0.3, 0.8) 10–12 years Model 1; OR = 0.9 (95%CI; 0.6, 1.2) Model 2; OR = 0.8 (95%CI; 0.5, 1.2)	Model 1:sex, and clustering by school only. Model 2:sex, physical activity, mother’s abd father’s weight status, matermal education, neightbourhood SES and clustering by school only.	Selection (★★★★) Comparability (★★) Outcome (★★★)
[11]b						Mother; 1108 Father; 947	-	-	BMI(OR)	Dog ownership and overweight or obese; Mothers; Model 1; OR = 1.2 (95%CI; 0.9, 1.5) Model 2; OR = 1.1 (95%CI; 0.9, 1.5) Fathers; Model 1; OR = 1.3 (95%CI; 0.96, 1.7) Model 2; OR = 1.1 (95%CI; 0.9, 1.5)	Model 1:clustering by school only. Model 2:physical activity, education, neightbourhood SES and clustering by school only.	Selection (★★★) Comparability (★★) Outcome (★★)
[29]	Pet ownership and blood pressure in old age.	Wright, J.D. et al, 2007	United States	Cross-sectional study	Pet	1,179 (354:825)	45.2: 41.0	64.3: 73.0	BMI (Mean)	Mean BMI; Non-pet owners; 25.4 Pet owners: 25.7	age	Selection (★★) Comparability (-) Outcome (★★)
[30]	To have or not to have a pet for better health?	Koivusilta, L.K. et al, 2006	Finland	Cross-sectional study	Pet	21,101 (8503:11,917)	39.4:42.1	Adult	BMI (%)	BMI distribution in pet owners and non-pet owners, respectively; ≥27; 26%, 21% <27; 74%, 79% Significant difference was observed by chi-square test (*p* < 0.011)	-	Selection (★★★★) Comparability (-) Outcome (★★)
[31]	Dog ownership, walking behavior, and maintained mobility in late life	Thorpe, Jr. R.J. et al, 2006	United States	Cross-sectional study	Dog	2533 (394:2137)	54.3:47.2	Non-dog owner (walked); 75.3 (2.9) Non-dog owner (did not walk); 75.8 (2.9) Dog owner (walked dog); 75.3 (2.6) Dog owner (did not walk dog); 75.3 (2.8)	BMI (%)	Obese (BMI >= 30); Non-dog owner (walked); 18.5 Non-dog owner (did not walk); 24.6 Dog owner (walked dog); 16.9 Dog owner (did not walk dog); 29.4	-	Selection (★★★) Comparability (-) Outcome (★★)
[12]	Pet ownership and risk factors for cardiovascular disease: another look	Parslow, R.A. et al, 2003	Australia	Cross-sectional study	Pet	5079 (2895:2184)	-	adult	BMI (OR)	Pet ownership and overweight; Age 40–44 years; OR = 1.30 (95%CI; 1.08, 1.55) Age 60–64 years; OR = 0.98 (95%CI; 0.83, 1.15) Pet ownership and obese; OR = 1.16 (95%CI; 1.00, 1.34)	age, sex and education or subgroups of these variables where appropriate.	Selection(★★★) Comparability(★★) Outcome(★★)
[32]	Pet ownership and risk factors for cardiovascular disease.	Anderson, W.P. et al, 1992	Australia	Cross-sectional study	Pet	5741 (784:4957)	59.1	20–59 years	BMI (Mean)	Mean (SD) of BMI in pet owners and non-petowners, respectively; Men; 25.4 (3.1), 25.5 (3.3), *p* = 0.64 Women; 24.2 (3.9), 23.9 (4.2), *p* = 0.29	-	Selection (★) Comparability (-) Outcome (★★★)

NHANES III, The Third National Health and Nutrition Examination Survey; BMI, Body Mass Index; OR, Odds Ratio; SD, Standard deviation; CI, Confidence Interval; CDC, Centre for Disease Control.

## Supplementary Materials

The following are available online at https://www.mdpi.com/1660-4601/17/10/3498/s1, Table S1: Search_String, Table S2: PRISMA 2009 checklist.
