# Supplementary material for: Association between Pet Ownership and Obesity: A Systematic Review and Meta-Analysis
Source: Int J Environ Res Public Health. 2020 May 17;17(10):3498. doi: 10.3390/ijerph17103498 (PMC7277191; doi:10.3390/ijerph17103498)
Supplement: Supplementary file 1 [file ijerph-17-03498-s001.zip › Table S1_Search_String.docx]

Suplementary 1-1: Search String used for identification of publications in PubMed

(((((("body mass index"[MeSH Terms] OR ("body"[All Fields] AND "mass"[All Fields] AND "index"[All Fields]) OR "body mass index"[All Fields]) OR ("obesity"[MeSH Terms] OR "obesity"[All Fields]) OR ("body size"[MeSH Terms] OR ("body"[All Fields] AND "size"[All Fields]) OR "body size"[All Fields]) OR ("waist circumference"[MeSH Terms] OR ("waist"[All Fields] AND "circumference"[All Fields]) OR "waist circumference"[All Fields]) OR ("overweight"[MeSH Terms] OR "overweight"[All Fields]) OR ("metabolic syndrome"[MeSH Terms] OR ("metabolic"[All Fields] AND "syndrome"[All Fields]) OR "metabolic syndrome"[All Fields]) OR ("adipose tissue"[MeSH Terms] OR ("adipose"[All Fields] AND "tissue"[All Fields]) OR "adipose tissue"[All Fields])) AND "humans"[MeSH Terms])) AND (("dog ownership" OR "cat ownership" OR "pet ownership" OR "pet owner" OR "cat owner" OR "dog owner"))) AND English[lang]) NOT obesity/veterinary

Supplementary 1-2: Search String used for identification of publications in Scopus

( ( ALL ( "body mass index" OR ( "body" AND "mass" AND "index" ) ) OR ALL ( "obesity" ) OR ALL ( "body size" OR ( "body" AND "size" ) ) OR ALL ( "waist circumference" OR ( "waist" AND "circumference" ) ) OR ALL ( "overweight" ) OR ALL ( "metabolic syndrome" OR ( "metabolic" AND "syndrome" ) ) OR ALL ( "adipose tissue" OR ( "adipose" AND "tissue" ) ) ) ) AND ( ALL ( "dog ownership" OR "cat ownership" OR "pet ownership" OR "pet owner" OR "cat owner" OR "dog owner" ) ) AND NOT ( ALL ( obesity AND veterinary ) ) AND ( LIMIT-TO ( EXACTKEYWORD , "Human" ) ) AND ( LIMIT-TO ( LANGUAGE , "English" ) )
